# A rare case of an incarcerated incisional hernia with gravid uterus

**DOI:** 10.1093/jscr/rjac571

**Published:** 2022-12-09

**Authors:** Xinlin Chin, Damon Li, Nicolas Copertino, Pamela J Caleo

**Affiliations:** Department of General Surgery, Sunshine Coast University Hospital, Birtinya, QLD, Australia; School of Medicine, Griffith University, Birtinya, QLD, Australia; Department of General Surgery, Sunshine Coast University Hospital, Birtinya, QLD, Australia; Department of General Surgery, Sunshine Coast University Hospital, Birtinya, QLD, Australia; Department of General Surgery, Sunshine Coast University Hospital, Birtinya, QLD, Australia

## Abstract

Herniation of a gravid uterus through an abdominal wall incisional hernia with overlying skin necrosis is exceptionally rare. A 29-year-old multiparous K30 + 4/40 pregnant female presented with a 1-month history of worsening abdominal wall skin changes. Magnetic resonance imaging of the abdomen and pelvis confirmed herniation of the gravid uterus into the hernia sac. A lower uterine segment caesarean section and hernia repair were performed by the general surgical and obstetrics team in view of the potential maternofoetal complications. This case highlights the importance of early recognition and the difficulties in managing gravid uterus herniation.

## INTRODUCTION

Herniation of a gravid uterus through an anterior abdominal wall hernia is an uncommon complication of caesarean section with potential incarceration and strangulation of the gravid uterus [1–3]. Planned caesarean section at term with simultaneous hernia repair is the recommended approach as hernia repair during pregnancy can be difficult and challenging [4]. We present a case of incarcerated gravid uterus herniation, the radiological findings, surgical management and review of literature.

## CASE REPORT

A 29-year-old multiparous (G5P4) K30 + 4/40 pregnant female was transferred from a peripheral centre with an irreducible incisional hernia containing her gravid uterus. Her previous four deliveries had been via lower segment caesarean section (LSCS) and aside from hernia formation were uncomplicated. The patient’s only other medical history was asthma.

The patient initially presented to her local Obstetrics unit at K26 with abdominal wall discolouration. Clinically, there was suspicion that the uterus had herniated through the abdominal wall and magnetic resonance imaging (MRI) confirmed this.

**Figure 1:**
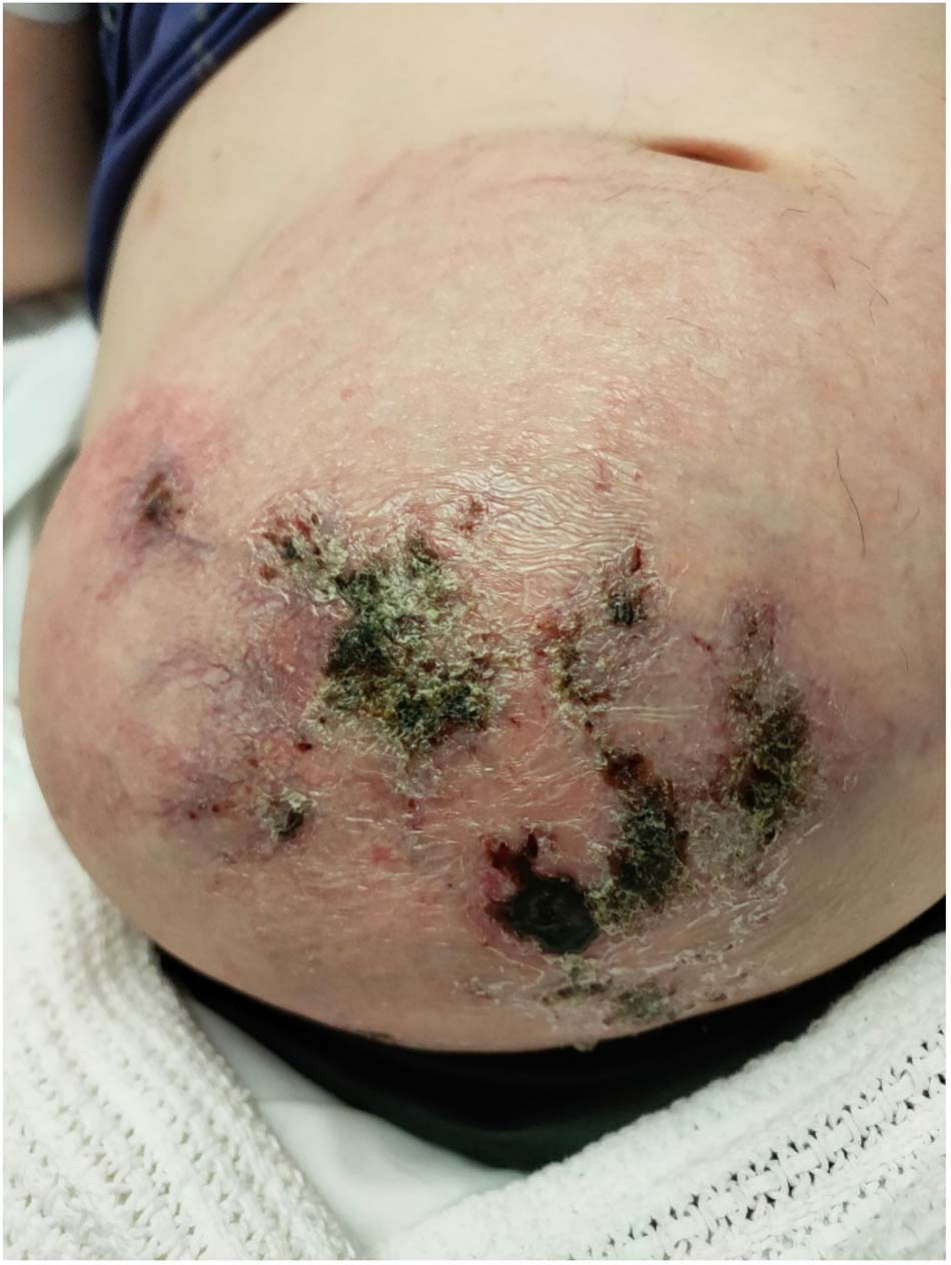
Gravid uterus herniating through the anterior abdominal wall incisional hernia with overlying skin necrosis at 30 weeks of gestation.

**Figure 2:**
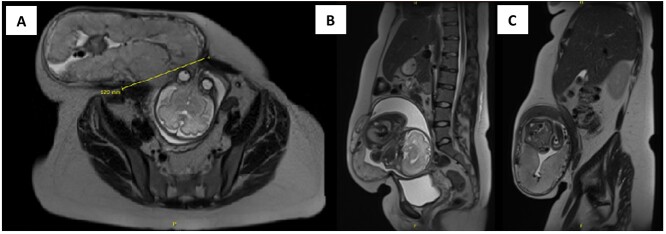
Axial (**A**) and sagittal (**B** and **C**) view of MRI of the abdomen and pelvis showing herniation of the gravid uterus into the right anterior abdominal wall with the neck of defect measuring 12 × 12 cm.

Upon presentation to our centre, the gravid uterus remained irreducible, but there was now ulceration and dry necrosis of the skin without evidence of infection (Fig. 1). Observations and routine bloods including inflammatory markers were unremarkable. The Obstetrics team had no immediate concerns for foetal well-being. Repeat MRI demonstrated herniation of the gravid uterus into the hernia sac, with a neck defect of 12 × 12 cm (Fig. 2).The placenta was positioned anteriorly, and superficially the uterus was noted to be within 2 mm of the skin.

With the progression of the pregnancy, there were concerns to both skin and uterine viability and ultimately the foetus. Accordingly, a semi-emergent case was planned at K30 + 6/40 conjointly between the General Surgical and Obstetrics teams.

**Figure 3:**
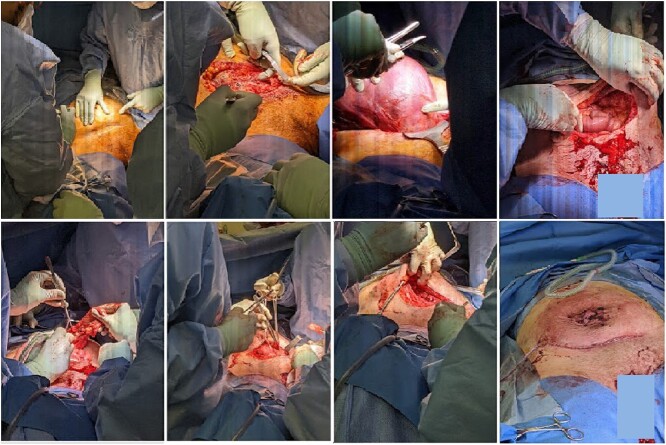
Intraoperative pictures of lower uterine segment caesarean section and abdominal wall hernia repair.

Pre-operative steroid loading was given in view of foetal prematurity. At operation, the foetus was delivered successfully via LSCS, the uterus was reduced into the abdominal cavity and the hernia sac was then excised completely. The abdominal wall defect was primarily closed in a vertical fashion with a continuous 0-PDS loop suture (Fig. 3). Such a repair was possible and tension-free due to the laxity of tissues from pregnancy. Mesh was avoided due to poor skin integrity, the high chance of repair failure and risk of contamination. A Blake’s drain was left in the subcutaneous space to manage potential seroma formation secondary to the large dead space. The overlying skin was left intact to allow demarcation in order to preserve skin for potential abdominoplasty.

The patient had an uneventful recovery and was discharged four days post-operatively. She was scheduled for surgical outpatient follow-up for consideration of formal hernia repair and abdominoplasty.

## DISCUSSION

Gravid uterus herniation is uncommon due to the size of the gravid uterus, which is often too large to enter the hernia sac when it reaches the level of the hernia neck [1, 3]. A review of the literature revealed that uterus herniation usually occurs between the gestational period of 4 to 8 months [1]. At present, there are only 21 reported cases of gravid uterus herniation through incisional hernias and 5 reported cases through umbilical hernias, in which 8 of them were incarcerated hernias with or without strangulation [1, 2, 5, 6].

Risk factors of incisional hernias include multiparity, obesity, post-operative pneumonia, poor surgical technique, inappropriate suture choice for rectus sheath closure, additional surgical procedures, wound infection and wound dehiscence [1, 3, 7]. Infraumbilical vertical incisions in caesarean sections give rise to most of the incisional hernias associated with uterus herniation as compared to transverse suprapubic incision due to the incision orientation and acting forces in the latter technique [3]. Our patient had previous transverse suprapubic incisions, however, was multiparous.

Gravid uterus herniation may lead to severe maternal and foetal complications including intrauterine growth restrictions, preterm labour, abortion, lower uterine segment rupture and poor placental perfusion causing foetal death [3, 7]. Increased pressure of the growing uterus in the hernia sac may cause vascular compromise of the overstretched skin, leading to skin ulceration, necrosis and complete evisceration of the gravid uterus [3–5]. Large skin ulcers with profuse bleeding may predispose the patient to developing anaemia and potential shock [1]. Uterine strangulation, which tends to occur in the late second trimester, warrants early hospital admission and caesarean section due to the potential risks [4, 7].

Management of herniation of the gravid uterus usually depends on the gestational age [3]. The success rate of conservative management with the use of abdominal binder and manual reduction is unknown [7, 8]. Patients with a herniated gravid uterus may still be able to undergo vaginal delivery successfully. Conservative management, elective caesarean section upon foetal maturity with simultaneous hernia repair and necrotic skin excision are recommended as the safest option due to concerns of abdominal wound integrity and risk of uterine rupture during vaginal delivery [1, 3–5, 8]. Abdominal wall incision needs to be planned with the aid of diagnostic imaging due to the possible presence of bowel in the hernia sac and thinning of the peritoneum and overlying skin [1]. Hernia repair with mesh can also be done in 6 to 8 weeks during the postpartum period as the risk of wound disruption will be lower due to abdominal wall changes during pregnancy [2, 8]. Tension-free mesh repair is the preferred method as it has a lower recurrence risk compared to suture repair regardless of the hernia size [3, 7]. Elective hernia repair during pregnancy is possible but generally avoided due to anaesthetic risk and high hernia repair failure rate due to ongoing uterus enlargement throughout the gestation period [2, 4].

The current management of gravid uterus herniation is guided by the patient’s clinical presentation, severity of complications and gestational age. As the hernia in our case was managed with a suture repair, a mesh repair may be required in the future if a recurrence were to occur. The number of cases of gravid uterus herniation may increase due to the increasing popularity of caesarean section; hence, its complications need to be discussed to facilitate early intervention.
